# Cross-reactivity between histone demethylase inhibitor valproic acid and DNA methylation in glioblastoma cell lines

**DOI:** 10.3389/fonc.2022.1033035

**Published:** 2022-11-16

**Authors:** Anna-Maria Barciszewska, Agnieszka Belter, Iwona Gawrońska, Małgorzata Giel-Pietraszuk, Mirosława Z. Naskręt-Barciszewska

**Affiliations:** ^1^ Intraoperative Imaging Unit, Chair and Department of Neurosurgery and Neurotraumatology, Karol Marcinkowski University of Medical Sciences, Poznan, Poland; ^2^ Department of Neurosurgery and Neurotraumatology, Heliodor Swiecicki Clinical Hospital, Poznan, Poland; ^3^ Institute of Bioorganic Chemistry, Polish Academy of Sciences, Poznan, Poland

**Keywords:** valproic acid, temozolomide, glioblastoma, DNA methylation, 8-oxo-deoxyguanosine

## Abstract

Currently, valproic acid (VPA) is known as an inhibitor of histone deacetylase (epigenetic drug) and is used for the clinical treatment of epileptic events in the course of glioblastoma multiforme (GBM). Which improves the clinical outcome of those patients. We analyzed the level of 5-methylcytosine, a DNA epigenetic modulator, and 8-oxodeoxyguanosine, an cellular oxidative damage marker, affected with VPA administration, alone and in combination with temozolomide (TMZ), of glioma (T98G, U118, U138), other cancer (HeLa), and normal (HaCaT) cell lines. We observed the VPA dose-dependent changes in the total DNA methylation in neoplastic cell lines and the lack of such an effect in a normal cell line. VPA at high concentrations (250-500 μM) induced hypermethylation of DNA in a short time frame. However, the exposition of GBM cells to the combination of VPA and TMZ resulted in DNA hypomethylation. At the same time, we observed an increase of genomic 8-oxo-dG, which as a hydroxyl radical reaction product with guanosine residue in DNA suggests a red-ox imbalance in the cancer cells and radical damage of DNA. Our data show that VPA as an HDAC inhibitor does not induce changes only in histone acetylation, but also changes in the state of DNA modification. It shows cross-reactivity between chromatin remodeling due to histone acetylation and DNA methylation. Finally, total DNA cytosine methylation and guanosine oxidation changes in glioma cell lines under VPA treatment suggest a new epigenetic mechanism of that drug action.

## Introduction

Valproic acid (2-propylvaleric acid, 2-propylpentanoic acid, VPA) is used primarily in the treatment of epilepsy, bipolar disorders neuropathic pain, and migraine prophylaxis. It is also a first-line antiepileptic drug (AED) for glioblastoma (GBM) patients (1). Although the mechanism of the VPA action is not fully understood, its action includes increased GABAergic activity, a reduction in excitatory neurotransmission, and modification of monoamines (2). VPA is mostly known due to its histone deacetylase (HDAC)-inhibitory activity (3), which is believed to be responsible for the antitumor action defined as epigenetic effects of this drug (4). Through the HDAC inhibition, VPA promotes transcription activity of chromatin, and a better access to transcription factors as well as the transcriptional machinery to DNA (5). Furthermore, it has been shown that VPA affects tumor cells by inhibiting proliferation, angiogenesis and promoting apoptosis. However, these activities on glioma cells are somewhat contradictory (4, 6, 7), and may allow more durable benefits from its anti-glioma properties.

**Figure 1:**
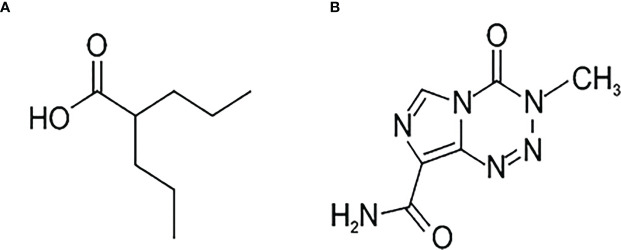
Chemical formulae of Valproic acid – **(A)** and temozolamide **– (B)**.

VPA (**Figure 1A**) is a branched short-chain fatty acid with a half-life of 9-16 h. Maximum plasma concentrations range from 25 to 100 mg/l following administration of 250 to 1000 mg dose. The protein binding capacity of VPA in plasma is approximately 90% in healthy persons. It is almost completely absorbed after oral administration, and dose-dependent peak plasma concentrations are attained within 1 to 10 hours. The bioavailability of VPA is nearly 100%, and its therapeutic concentration is 50–100 µg/ml (340–700 µM/l) (8). However, VPA penetration through the blood–brain barrier (BBB) changes because of an asymmetric transport of valproate, such that the brain-to-blood flux exceeds the blood-to-brain flux. Only a free, non-protein-bound, portion of VPA crosses the BBB and shows its antiepileptic effect (9). Valproate concentration in the cerebral cortex is remarkably low, compared with either total or unbound VPA concentration in plasma. The respective brain-to-serum partition ratios based on the total and free drug in serum are 0.11 ± 0.05 and 0.54 ± 0.18, respectively (10). Other studies showed brain levels of VPA at 6.8-27.9% and CSF levels at 7.6-25.0% of the plasma level (11). Altogether, understanding the mechanism of action of VPA is necessary.

The best known chemotherapeutic agent for GBM is temozolomide (TMZ). Temozolomide, (4-methyl-5-oxo-2,3,4,6,8-pentazabicyclo[4.3.0]nona-2,7,9-triene-9- carboxamide) (**Figure 1B**) is an oral alkylating agent that significantly prolongs survival in GBM patients when administered during and after radiotherapy (12). It is used as first-line chemotherapy, but also shows significant activity against recurrent glioblastoma (13). TMZ is available to the central nervous system (CNS) because its lipophilic properties allow efficient crossing of the BBB. TMZ interferes with the mechanism of development of cancer, slowing down its growth and spread in the body. O^6^-methylguanine (O^6^-mG) is regarded as the primary cytotoxic lesion of TMZ in DNA, although, it constitutes only ca. 5% of the TMZ-mediated methylation reaction products. Therefore, it is not clear why methylation of the O^6^ position of guanine can be the major player in cytotoxic drug action.

Glioblastoma is the most frequent, highly recurrent, and rapidly progressing type of astrocytic brain tumor in adults. It is characterized by uncontrolled proliferation, dynamic angiogenesis, invasiveness, and the ability to evade apoptosis and infiltrate the neighboring brain. There are much data showing that GBM is mainly driven by genetic and epigenetic aberrations (14). The standard therapy for glioblastoma patients consists of surgical resection followed by radiation therapy and concomitant chemotherapy with temozolomide (12). However, even with that therapeutic scheme, the median survival time of GBM patients is still below 12 months from diagnosis, and the 5-year survival rate is less than 5%. Therapy failure, observed in the vast majority of glioblastoma patients, and bad prognosis are probably due to high intrinsic resistance to chemo- and radiotherapy on the one hand, as well as the rapid spread of glioblastoma cells in the brain on the other. Therefore, a new strategy to treat GBM is still urgently needed. Up to 50% of glioblastoma patients develop tumor-associated epileptic seizures that are treated with AEDs. Glioma patients with a history of seizures have a better prognosis than patients without seizures, and it has been reported that this phenomenon could be related to the VPA used for seizure prophylaxis or treatment (15). It has been shown that VPA might have a synergistic antitumor effect with radiation therapy because of the radio-sensitizing properties of VPA (16). On the other hand, there are clinical trials suggesting that the addition of VPA to the standard radiotherapy with temozolomide in newly diagnosed glioblastoma patients may prolong survival (17), but others show no significant difference in overall survival (18). In addition to that, a meta-analysis confirmed some survival benefits unequally (19). Even mechanism of VPA action is vague and the impact on GBM patients’ survival is doubtful, still VPA is the most commonly used AED during glioblastoma treatment. Therefore, we decided to look more precisely at the mode of action, of this epigenetic drug.

Epigenetics provides a new explanatory area for many pathological processes. It offers a connection between genetic and environmental factors that influence the development of the disease. Epigenetic regulation of gene expression is a dynamic, responsive, and reversible process. It plays a critical role in the pathophysiology of diseases, from neurological to metabolic disorders, to cancer and rare diseases thus leading to the tailoring of conventional therapies and ultimately better outcomes. DNA methylation is an essential mechanism for gene expression regulation, genomic imprinting, development, and genomic stability. DNA methylation status can significantly alter transcription factor binding and is thought to be inversely proportional to the level of expression of gene products. The bestcharacterized epigenetic marker is 5-methylcytosine (m^5^C) in DNA (20). Therefore, the implications of DNA methylation changes are numerous (21, 22). Recently, we have shown that m^5^C is a diagnostic marker for brain tumors’ malignancy and the severity of other diseases (23, 24). In other studies we have found that temozolomide which modifies DNA through methylation (damage) of oxygen 6 of guanosine in DNA, affects methylated cytosine (m^5^C) level, causing oxidative demethylation (25, 26).

Another DNA damage product is 8-hydroxydeoxy guanosine (8-oxo-dG), formed by the reaction of guanosine residue with hydroxyl radical (•OH). It induced base paring not only with cytosine (Watson-Crick base pair) but also with adenosine causing G-T transversion. A high level of 8-oxo-dG as a result of oxidative stress contributes to genome instability, elevated proliferation rate, and metastasis. A mutual monitoring of m^5^C and 8-oxo-dG level in DNA will provide data for discussion of VPA effects on the cell.

The aim of the present work was to look for a new mechanism of VPA action in glioblastoma cells. We showed that it works not only as HDAC inhibitors but also as an effector of DNA methylation. It is known that epigenetic modifications respond to environmental changes more rapidly than genetic ones. Therefore, the level of their changes seems to be most promising in the treatment of such diseases as cancer.

In the paper, we showed that VPA stimulates DNA cytosine methylation, and did not change significantly red-ox potential of the GBM cells. The effect of combined treatment of VPA and TMZ is toxic to the cell.

## Materials and methods

### Chemicals and reagents

Sodium valproate (Sanofi-Aventis, France) stock solution of 100 mg/ml in water was used to prepare the required concentration with a complete medium. Temozolomide (Merck, Germany) was dissolved in dimethyl sulfoxide (DMSO, Sigma) at a concentration of 0.103 M. [γ-P^32^] ATP (6000 Ci/mmol) was purchased from Hartmann Analytic GmbH (Germany), T4 polynucleotide kinase was purchased from USB (UK), micrococcal nuclease, spleen phosphodiesterase II, apyrase, RNase P1, thiazolyl blue tetrazolium bromide, and inorganic salts were purchased from Sigma Aldrich/Merck (Germany), cellulose plates and methanol were purchased from Merck (Germany), and the Genomic Mini kit for DNA isolation was supplied by A&A Biotechnology (Poland).

### Cell line and culture conditions

The human glioblastoma cell lines (T98G, U138, U118), the cervical cancer cell line (HeLa), and the human keratinocyte cell line (HaCaT) were purchased from ATCC (USA). T98G and U138 cell lines were cultured in EMEM medium (ATCC), U118 in DMEM (ATCC), HeLa, and HaCaT in EMEM (Sigma-Aldrich). Each medium was supplemented with 10% (v/v) fetal bovine serum (FBS, Sigma-Aldrich) and 10 mg/ml antibiotics (penicillin 100 U/ml and streptomycin 100 μg/ml). Cells were cultured at 37°C with 5% CO_2_ in humidified air. After 24* h*, cells were washed with phosphate-buffered saline (PBS, Sigma-Aldrich/Merck), placed in a fresh medium, and treated with VPA alone or a mixture of VPA and TMZ.

### Cell viability/proliferation assay

Cell viability was evaluated with a dye-staining method, using 3-(4,5-dimethyl-2-thiazolyl)-2,5-diphenyl-2H-tetrazolium bromide (MTT) (27). Cell lines (HaCaT, HeLa, T98G, U118, U138) were seeded in 96-well culture plates at a density of 1×10^4^ cells/well and grown in the supplemented medium at 37°C under 5% CO_2_ atmosphere. Next the cell lines were treated with VPA at concentrations (20, 39, 78, 156, 313, 625, 1250, 2500, 5000, 10000, 20000 µM). The combinations of TMZ concentrations of 0, 1, 30, and 100 µM with the above mentioned VPA concentrations were performed to show the concomitant effect of dual treatment. After 24* h*, the supernatant was washed out, and 100 µl of MTT solution in medium (0.5 mg/ml final concentration of MTT) was added to each well for 2* h*. After the incubation, the unreacted dye was removed through aspiration. The formazan crystals were dissolved in 100 µl/well DMSO and measured spectrophotometrically in a multi-well Synergy2 plate reader (BioTek Instruments, USA) at a test wavelength of 492 nm and a reference wavelength of 690 nm. The half-maximal inhibitory concentrations (IC_50_) were calculated by fitting experimental values to the sigmoidal bell-shaped equation using GraphPad Prism v5.01 (GraphPad Software, Inc., USA). Values represent the means from three independent experiments.

### Treatment of the cell lines with valproic acid

VPA stock solution was added directly to the culture medium (with 90–95% confluence) to get the required concentrations (30, 50, 100, 250, 500 μM) and incubated for 3, 12, 24, and 48* h*. For the control reaction the cells were treated with H_2_O only. After 3–48 h of VPA treatment, cells were washed with PBS, trypsinized, and collected by centrifugation at 4000 rpm for 10* min*. The cellular pellets were quickly frozen and stored at 20°C for DNA isolation.

### Treatment of the cell lines with the combination of valproic acid and temozolamide

VPA and TMZ stock solutions were added directly to the culture medium to get the designed concentration. In experiments with TMZ, the final DMSO concentration in each cell culture was 0.8%. Cell cultures (with 90–95% confluence) were washed with PBS and placed in fresh medium, and treated with: 50 μM VPA and 0, 1, 30, or 100 μM TMZ for 3, 24 and 48* h*; 200 μM VPA and 0, 1, 30, or 100 μM TMZ for 3, 24 and 48* h*; 350 μM VPA and 0, 1, 30, or 100 μM TMZ for 3, 24 and 48* h*. The control cells were treated with H_2_O (for VPA) and DMSO (for TMZ). After incubation, the cells were washed with PBS, trypsinized, and collected by centrifugation at 4000 rpm for 5* min*. The cellular pellets were quickly frozen and stored at 20°C for DNA isolation.

### DNA isolation from cell cultures

DNA from tissue samples was extracted with the Genomic Mini kit according to the manufacturer*’*s instructions. Shortly, tissue samples were incubated with RNase A first and then with proteinase K. After centrifugation (15000 rpm for 3* min*), the supernatant was applied to a mini-column, and DNA bound to the column was eluted with Tris-buffer pH 8.5 and stored at 20°C for further analysis. The purity of DNA preparations was assessed by measuring UV absorbance at 260 and 280 nm. The A_260_/A_280_ ratio was 2.0–2.1.

### Analysis of m^5^C contents in DNA

DNA (dried, 1μg) was dissolved in a succinate buffer (pH 6.0) containing 10 mM CaCl_2_ and digested with 0.001 units of spleen phosphodiesterase II and 0.02 units of micrococcal nuclease in 4 μl total volume for 5 h at 37*°*C. DNA digest was labeled with 1μCi [γ-P^32^]ATP (6000 Ci/mmol) and 1.5 units of T4 polynucleotide kinase in 10 mM bicine-NaOH pH 9.7 buffer containing 10 mM MgCl_2_, 10 mM DTT, and 1 mM spermidine. After 0.5* h* at 37*°*C, apyrase (10 units/ml) in the same buffer was added and incubated for another 0.5 h. The 3*’*nucleotide phosphate was cleaved off with 0.2 μg RNase P1 in 500 mM ammonium acetate buffer, pH 4.5. Identification of [γ-P^32^]m^5^C was performed with two-dimensional thin-layer chromatography (TLC) on cellulose plates using the solvent system isobutyric acid:NH_4_OH:H_2_O (66:1:17 v/v) in the first dimension and 0.2 M sodium phosphate (pH 6.8)-ammonium sulfate-n-propyl alcohol (100 ml/60 g/2 ml) in the second dimension. Radioactivity was subsequently measured using a Fluoro Image Analyzer FLA-5100 with Multi Gauge 3.0 software (FujiFilm). Each analysis was repeated three times. For precise calculations, we evaluated spots corresponding not only to m^5^C, but also to products of its degradation, such as cytosine (C) and thymine (T). The amount of m^5^C was calculated as R = [(m^5^C/m^5^C+C+T)]*100 (23).

### Analysis of 8-oxo-dG contents in DNA

DNA was dissolved in 200 µl of a buffer (pH 5.3) containing 40 mM sodium acetate and 0.1 mM ZnCl_2_, then mixed with nuclease P1 (Sigma-Aldrich, St. Louis, MO, USA) solution (30 µg), and incubated for 3 h at 37°C. Then, 30 µl of 1M Tris–HCl pH 8.0 and 5 µl of alkaline phosphatase (1.5 units) solution was added, followed by 1 h incubation at 37°C. DNA hydrolysate was purified using a cut-off 10,000 Da filter. 8-oxo-dG amount in DNA was determined using HPLC (Agilent Technologies 1260 Infinity, CA, USA) with two detectors working in series: 1260 Diode Array Detector and Coulochem III Electrochemical Detector (ESA Inc., Chelmsford, MA, USA). Isocratic chromatography of DNA hydrolysate was performed using a solution of 50 mM CH_3_COONH_4_ at pH 5.3 and methanol (93:7). Analysis of dG for reference was performed at 260 nm. 8-oxo-dG was determined with the following electrochemical detection settings: guard cell +400 mV, detector 1: +130 mV (screening electrode), detector 2: + 350 mV (measuring electrode set on the 100 nA sensitivity) (28).

### Calculation of the genomic amount of m^5^C and 8-oxo-dG in human DNA

The amount of modified bases in DNA was calculated on the basis of global genome composition - 3.05× 10^9^ bases (100%), where C - 624 × 10^6^ (20,5%), T - 905 × 10^6^ (29,6%), G - 623 × 10^6^ (20,4%), A - 901 × 10^6^ (29,5%), and m^5^C - 31 × 10^6^ (1%). The amount of m^5^C (%) in pyrimidines in DNA was determined from TLC analysis with the formula R[%] = m^5^C × 100/(C + T). The total number of m^5^C in the genome was calculated from the formula m^5^C = (1 498 333 975) × R/100. The input amount of guanosine was necessary to determine 8-oxo-dG contents. It was calculated from Diode Array Detector (PAD) measurements using Avogadro number N_G_ = 6.02 × 10^20^ × b(mAU)/a(mAU) standard. 8-oxo-dG nucleoside amount was estimated with electrochemical detector N_8-oxo-dG_ = 6.02 × 10^20^ × d(nA)/c(nA) standard. The total number of 8-oxo-dG = 623 × 10^6^ × N_8-oxo-dG_/N_G_ (29).

### Statistical analysis

STATISTICA software, (StatSoft Polska), was used for analyses of all data, as previously (30). The data are the result of three independent experiments. The descriptive statistics function was used to generate the mean and SD. The one-tailed t-test was used to calculate significant differences in R values for tested samples as compared with the control. Standard deviations were indicated as error bars on graphs.

## Results

### Cytotoxic effect of VPA in neoplastic and normal cell lines

**Figure 2:**
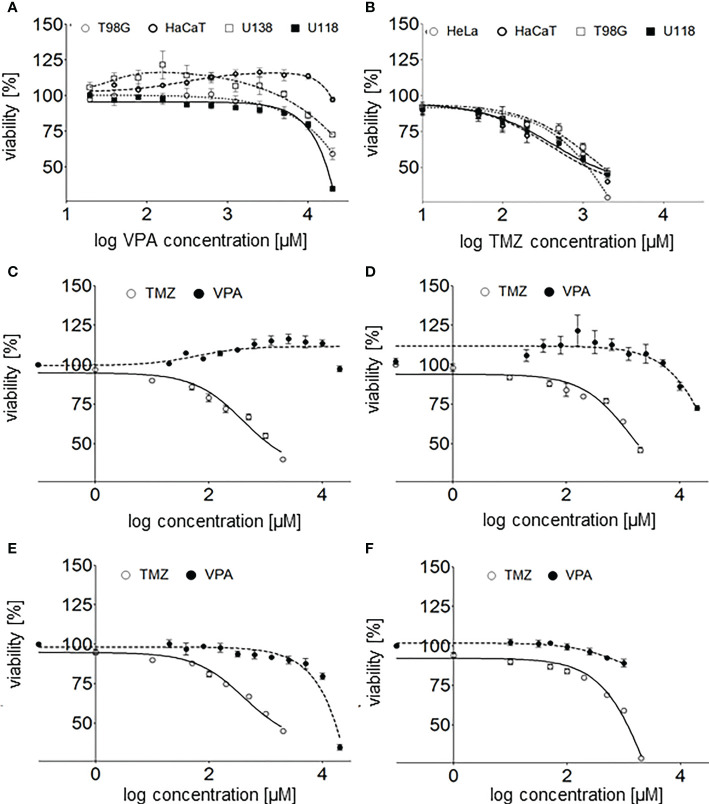
**(A) -** valproic acid’s effect on human glioblastoma cell lines (T98G, U118, and U138) and a keratinocyte cell line (HaCaT) viability 24 h after cell culture supplementation with VPA (20-20000 µM; log concentration, 1.3 - 4.3 µM). **(B)** - TMZ’s effect on human glioblastoma cell lines (T98G, U118), a keratinocyte cell line (HaCaT) and an adenocarcinoma cell line (HeLa) viability 24 h after cell culture supplementation with TMZ (1 µM - 2 mM, log concentration 0–3.3 µM). The comparison of TMZ and VPA effect on the viability of **(C)** - U118, **(D)** - T98G, **(E)** - HaCaT, and **(F)** - HeLa cells. Values are the means ± SE of at least four experiments.

Cell viability was determined by MTT assay at concentrations in the range of 20 µM–20 mM (1.29 - 4.3 on the logarithmic scale) and 1 µM–2 mM (0 - 3.301 on the logarithmic scale) for VPA and TMZ, respectively. VPA below 1000 µM (3 on logarithmic scale) showed no effect either on U118 or T98G glioma cells (**Figures 2A, D, E**), in a range 1 000 – 10 000 µM (3 – 4 on logarithmic scale) slight, and above 10 000 µM (4 on logarithmic scale) significant decrease of these cells viability

Interestingly, no toxicity of VPA at concentrations below 5 000 µM (3.7 on logarithmic scale) toward U138 and HaCaT cells was observed. What is more, a slight increase in cells viability in a range of 40 - 1 250 µM (1.6 – 3.1 on logarithmic scale) and 625 – 10 000 µM (2.8 – 4 on logarithmic scale) VPA was reported for U138 and HaCaT cells, respectively (**Figures 2A, C**). TMZ is much more toxic for all tested cell lines than VPA (**Figures 2C–F**). TMZ at concentrations above 50 μM shows a significant decrease in the viability of human glioblastoma cell lines (T98G, U118), a keratinocyte cell line (HaCaT), and a cervical cancer cell line (HeLa) (**Figures 2B–F**). Calculated IC50 for T98G and HaCaT cell lines were 2.3 and 1.2 mM, respectively (31, 32).

Combination index (CI) of VPA and TMZ was calculated with CompuSyn software (31, 32), for T98G cells, treated simultaneously with different concentrations VPA (up to 20 mM) and 100uM TMZ or VPA only. Clearly combination index, CI_VPA-TMZ_ value is above >1 (IC = 2.55, for fa = 0.5), which suggests antagonistic effect of these drugs on cell viability.

### The effect of VPA on genomic DNA methylation level in cell lines

**Figure 3:**
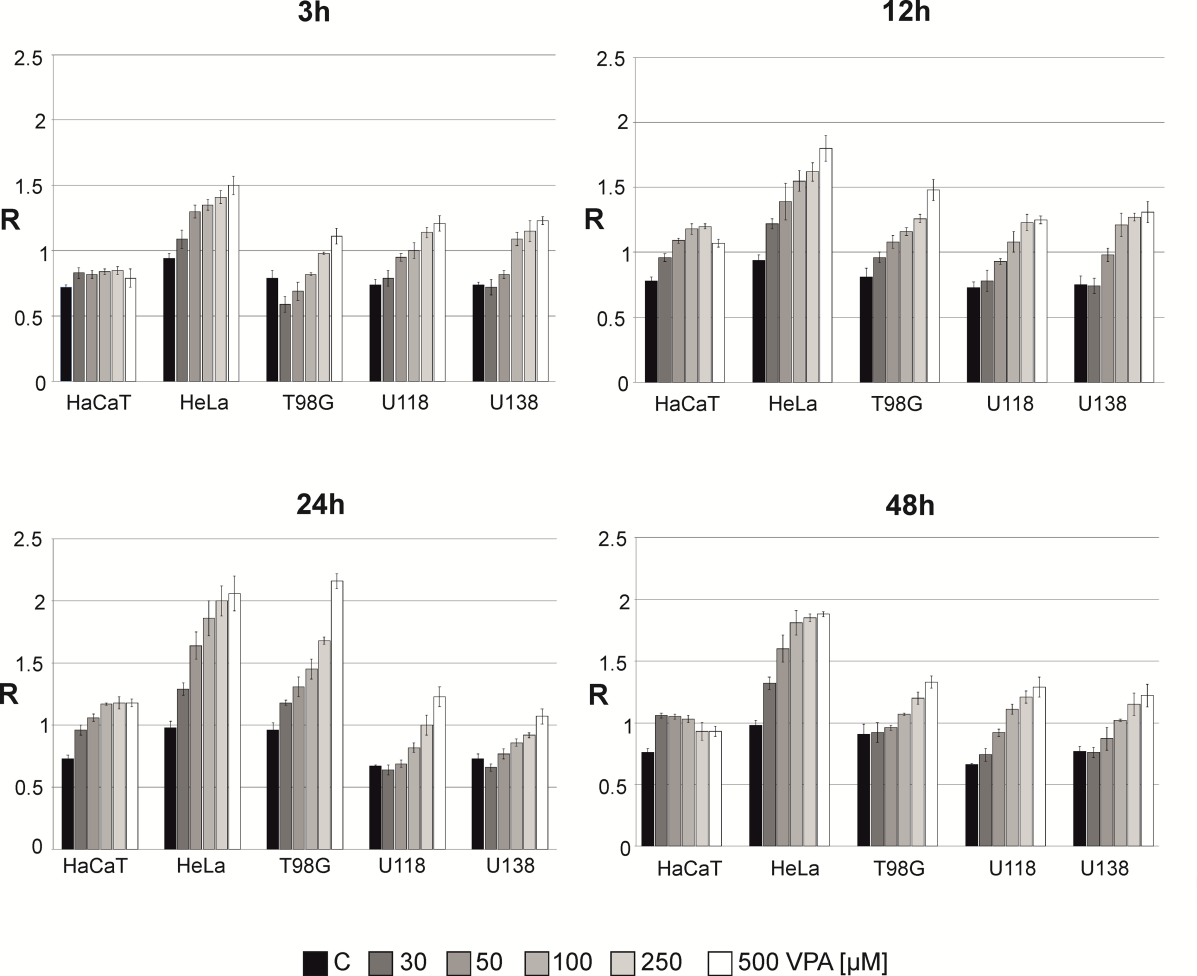
The effect of valproic acid (VPA) on DNA (m^5^C) methylation level (R) in different cell lines. The analysis was performed after 3, 12, 24, and 48 h of incubation in a given VPA concentrations (0, 50, 100, 250, 500 μM). For control experiment cells were treated with H_2_O. The R values are means from three experiments.

The total DNA methylation level (expressed as R) in the cell lines was analyzed in after treatment with 30-500 μM of VPA for 3-48* h* (**Figure 3**). We noticed concentration and time dependent m^5^C contents changes. Small fluctuations of DNA methylation were observed for a normal cell line (HaCaT), where are m^5^C level increased slightly for other cell lines after 12 and 24* h* of incubation. For the HeLa cell line we observed the highest increase in total DNA methylation. For glioblastoma cell lines at the concentrations of 250-500 μM (for U138 and U118 also in 100 μM), we noticed that m^5^C content increased in a dose-dependent manner. For T98G, a significant increase in DNA methylation at all concentrations was visible after 24* h*. However, a longer incubation time (48* h*) showed a lower level of m^5^C, which suggests demethylation (**Figure 3**). The differences in R values between different VPA concentrations in all cell lines were statistically significant (**Supplementary Table S1**).

### The effect of VPA and TMZ on genomic DNA methylation level in cell lines

**Figure 4:**
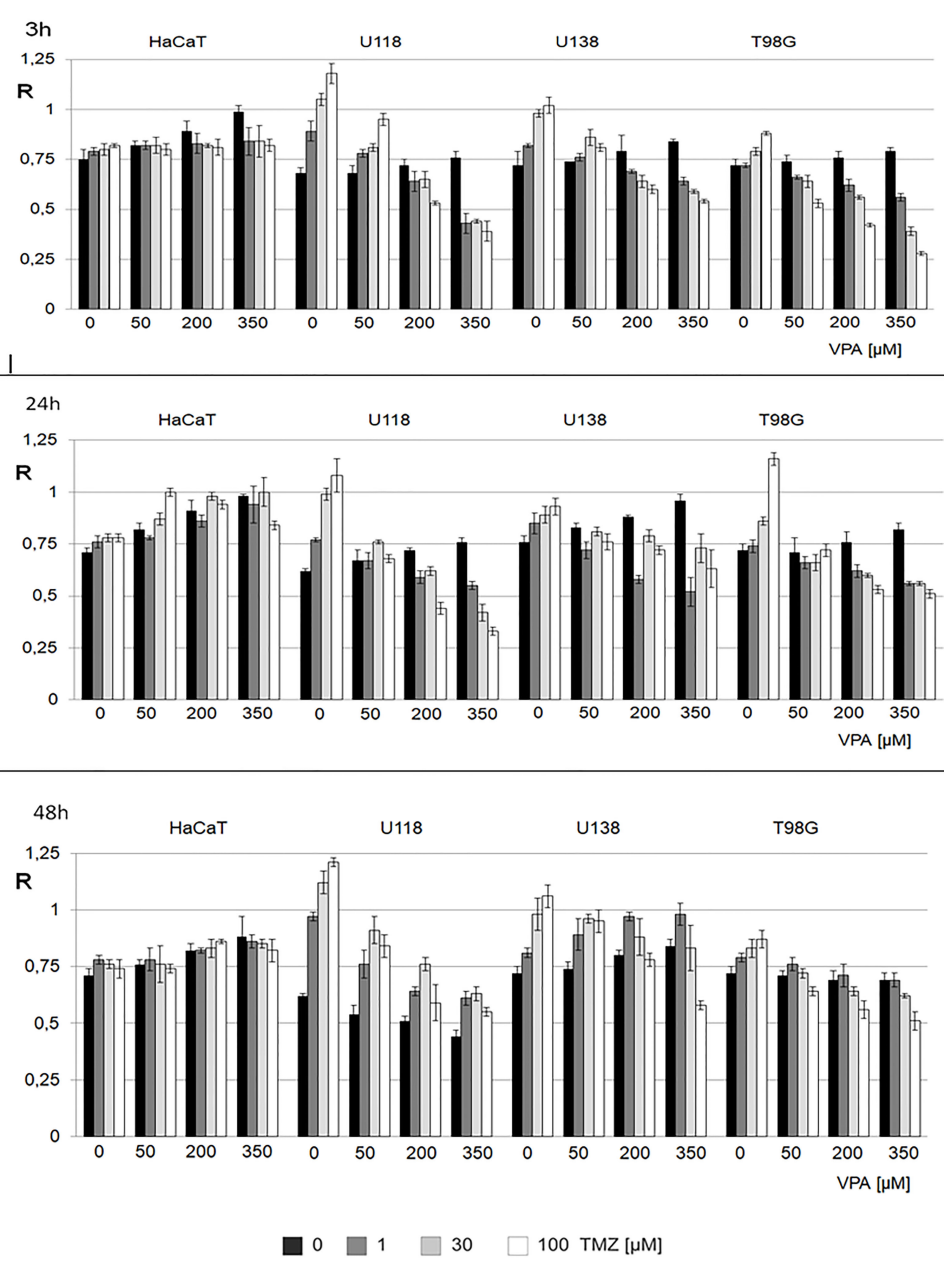
The effect of action of VPA/TMZ on total DNA (m^5^C) methylation level in different cell lines. The analysis was performed after 3, 24 and 48 h of incubation in a given VPA (0, 50, 200, 350 μM) and TMZ concentration (0, 1, 30, 100 μM). For control experiments cells were treated with DMSO only. The R values are means from three experiments. The reference (100%) is the viability of cells not treated with TMZ and VPA. Values are means from three experiments.

We analyzed the combined effect of VPA (0, 50, 200, and 350 μM) and of TMZ (0, 1, 30, and 100 μM) for 3, 24, and 48* h* on human glioblastoma (T98G, U138, U118) and keratinocyte (HaCaT) cell lines (**Figure 4**). In control experiments, cell lines were treated with H_2_O and DMSO for VPA and TMZ, respectively. The effect of VPA/TMZ action on glioblastoma cell lines is variable. One can see VPA alone produces a clear dose-dependent increase in genomic DNA methylation in glioblastoma cell lines and scarcely in a non-neoplastic cell line. For HaCaT cell line, longer incubation (24* h*) was crucial to obtain a statistically significant increase in total DNA methylation with VPA and TMZ (**Supplementary Table S2**). On the other hand for T98G, U138, and U118 cell lines, TMZ catalyzes the removal of m^5^C, which clearly proves DNA demethylation. The decrease in m^5^C contents in the DNA of T98G cell line is negatively correlated with treatment with TMZ (100 μM) and VPA (350 μM), for 3* h* (**Figure 4**). Similar observations can see for U118 for 24h (**Figure 4**). A slightly higher increase in m^5^C contents induced with the increasing amount of TMZ was observed only for the U138 cell line at 50 μM of VPA after 48* h* of incubation. The differences in R values are statistically significant (**Supplementary Table S2**).

### Cytotoxic effect of a combination of VPA and TMZ in neoplastic and normal cell lines

**Figure 5:**
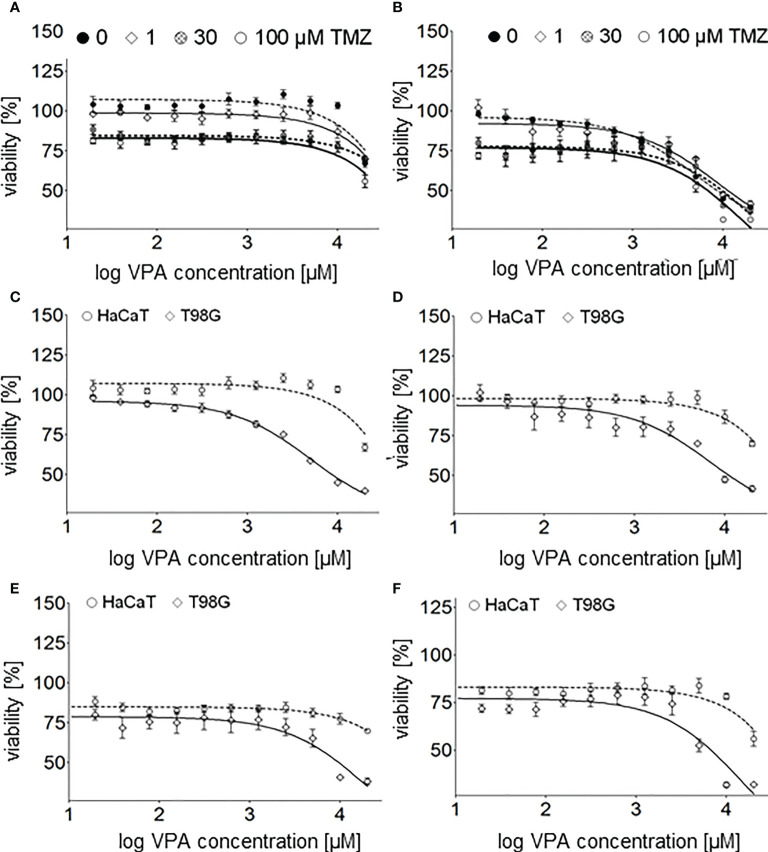
Cell viability assay of the glioblastoma cell line T98G – **(A)**, and the keratinocyte cell line HaCaT – **(B)** 24 h after cell culture supplementation with both TMZ (0, 1, 30, 100 µM) and different concentrations (20, 39, 78, 156, 313, 625, 1250, 2500, 5000, 10000, 20000 µM) of VPA. Cell viability assay of the glioblastoma cell line T98G and the keratinocyte cell line HaCaT 24 h after cell culture supplementation with different concentrations (20–20000 µM, log concentration 1.3 - 4.3 µM) of VPA – **(C)** and both VPA and 1 µM – **(D)**, 30 µM – **(E)** and 100 µM - **(F)** of TMZ. Values are means ± SE of at least four experiments.

Cell viability of glioblastoma (T98G) and keratinocyte (HaCaT) cell lines undergoing combined therapy was determined with MTT assay in the range of 20 µM - 20 mM (1.3 – 4.3 on logarithmic scale) VPA and 1, 30 and 100 µM (0, 1.5 and 2 on logarithmic scale TMZ (**Figure 5**).

T98G cells seem to be more sensitive than HaCaT to TMZ (**Figures 2B**, **5D–F**), VPA (**Figure 2A**) and a combination of TMZ and VPA (**Figures 5A–F**). VPA, in a concentration range of 625 – 10 000 µM (2.8 – 4 on logarithmic scale), increases the viability of HaCaT cells (**Figures 2A**, **5A, C**). Such an effect was observed (but is much lower) in the case of HaCaT cells treated already with TMZ (**Figures 5A, D–F**). It means that VPA in such concentrations protects healthy cells (HaCaT) from cell death being a result of TMZ (30 or 100 µM). In the case of T98G, in the range of 20 µM - 10 mM VPA no additional toxicity of T98G cells treated already with TMZ was observed (**Figures 5B, D–F**). However, VPA above 625 µM (2.8 on logarithmic scale), excelerates the cytotoxic effect of TMZ on T98G cells (**Figures 5B, D–F**).

### Analysis of m^5^C and 8-oxo-dG contents in DNA of T98G cell lines treated with VPA and TMZ

**Figure 6:**
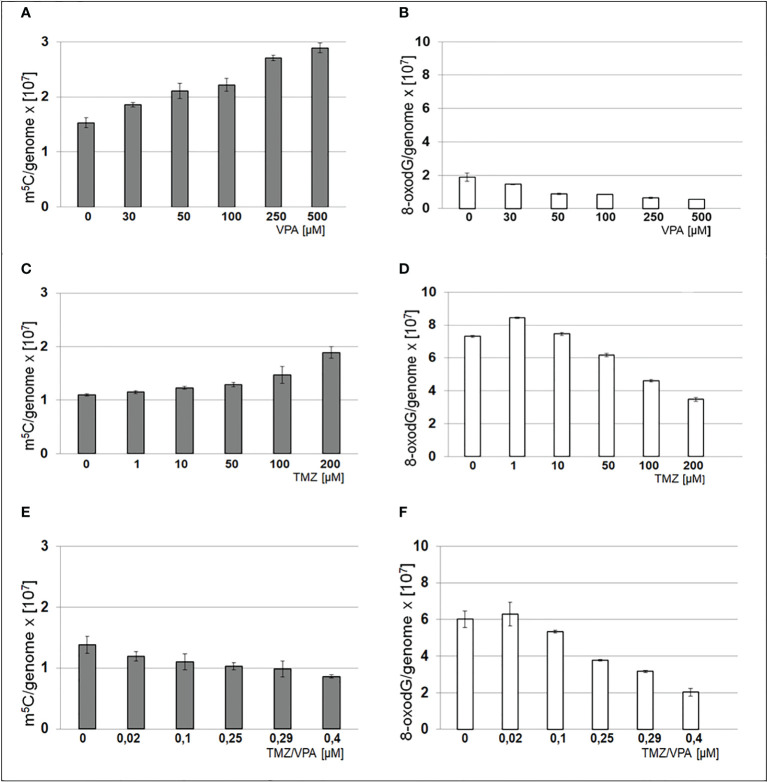
Contents of m^5^C – **(A, C, E)** and 8-oxo-dG – **(B, D, F)** in DNA from T98G cell line after treatment with TMZ, VPA or their mixture. The analysis was performed after 24 h of incubation in given TMZ (0, 1, 10, 50, 100 and 200 μM), and VPA (0, 30, 50, 100, 250, 500 μM) concentration and mixtures of both of TMZ/VPA at ratio (0, 0.02, 0.1, 0.25, 0.29, 0.4). In control experiment cells were treated with DMSO only. The R values are means from three experiments.

The total level of m^5^C and 8-oxo-dG in genomic DNA from the T98G cell line after treatment with TMZ, VPA, and their mixture was analyzed. One can see that treatments with TMZ and VPA separately, show an increase of m^5^C contents and a decrease of the 8-oxo-dG amount in DNA (**Figure 6**). On the contrary combined TMZ and VPA action induced cellular stress and DNA cytosine hypomethylation, and small changes in 8-oxo-dG. A decrease in the amount of 8-oxo-dG suggests the induction of DNA repair mechanisms.

## Discussion

In a search for an understanding of the mechanism of VPA action, we analyzed changes in the total genomic contents of 5-methylcytosine, the main epigenetic modification of DNA in cell lines. The reason for focusing on that marker is that the DNA methylation (m^5^C) profile changes dynamically under the influence of environmental, nutritional, and pathogenic conditions, viruses, and many other factors, as well as development and aging (33). The presence or absence of DNA (m^5^C) methylation functions as a “switch”, repressing or activating gene transcription, respectively, and therefore providing an essential mechanism for tissue-specific, developmentally regulated, and environmentally influenced genetic processes (14). In parallel to the analysis of m^5^C, we also looked at 8-oxo-dG level in DNA, which is well known DNA oxidative damage marker.

In the present study, we analyzed the influence of VPA on genome wide methylation. It turned out that VPA alone increases total DNA methylation in a dose-dependent manner. We adjusted VPA doses to those virtually achieved in the central nervous system during treatment (11). While therapeutic serum concentrations of VPA are 340–700 µM, its brain concentration is approximately 7-30% of that in plasma. Therefore, to analyze the real VPA impact on the central nervous system, one should focus on the VPA concentration in the range between 23 and 490 µM. Therefore, in our experiments, we used a concentration of 30-500 µM. VPA concentration of 1 mM is already double our maximum. Based on our previous observations with high doses of TMZ, one can presume that in such high concentrations, DNA demethylation can be an effect of oxidative stress and DNA modification as well as induction of the DNA repair mechanism (25). However, DNA demethylation in VPA-treated HeLa cell lines has been observed (34) although that analysis has been done with FT-IR microspectroscopy, at high VPA concentration (1 and 20 mM) for 24 h. In another study, the glioma stem cell colonies were treated with 2 mM VPA for 24 h and up to 30 days, and TMZ of 50-400 μM for 48 or 72 h. VPA in that conditions, does not increase TMZ efficacy (35). This is understandable in the scope of our observations, showing that the joining of VPA and TMZ action causes global DNA demethylation (**Figure 4**). Interestingly it has been previously shown that VPA downregulates MGMT expression in glioma cells (36). In the case of 0.5–10 μM TMZ, DNA hypomethylation in glioblastoma cell lines depends on the cell line. This effect is particularly interesting because that range of concentration is observed in the brain during TMZ treatment (26). At the concentration of 10 μM of TMZ (3 μM in the case of U118), dose-dependent DNA hypermethylation was observed. The hypomethylating effect of VPA/TMZ treatment is most striking after 3 h and is more balanced after a long time of incubation (24 and 48 h) (**Figure 4**). It has been frequently observed for different environmental pollutants (37). Comparing the results for drug combination (**Figures 6C–F**) with the single drug effects (**Figures 2D, E**), one can say that VPA shows a positive effect on longer cells’ viability under TMZ treatment. Several mechanisms underlying the anti-tumor effect of VPA have already been considered (38). One of them regards VPA as histone deacetylase (HDAC) inhibitor, which increases lysine acetylation on histones, as well as other nonhistone proteins, by downregulating the HDAC activity. Removal of positive charge from lysine residue induces loosening of the chromatin structure and provides better accessibility to transcription factors to their target DNA sequence (39). Therefore, HDAC inhibitors induce numerous downstream effects such as cell cycle arrest, induction of apoptosis, affection of angiogenesis, inhibition of cellular stress response pathways, and changing ncRNA expression (39). However, VPA is a relatively weak HDAC inhibitor at millimolar concentrations, and is only an active inhibitor at relatively high concentrations (40). It actually excludes that mechanism from place in the brain because of the very small penetration of the drug through BBB. One should remember that HDAC inhibitors can reverse CpG methylation by the down-regulation of DNMT1 expression or by the repression of ERK1, and gene silencing (41). Those observations provide support for our concept of the novel epigenetic mechanism of VPA action which stimulates genome-wide DNA methylation. A possible reason for that can be drug-induced DNA hypermethylation (42). 5-methylcytosine is a product of an enzymatic reaction catalyzed by DNA methyltransferases (DNMTs), where S-adenosyl methionine (SAM) is the only methyl donor. VPA is not a substrate for DNMTs, therefore, the only possible mechanism where a drug can increase m^5^C contents is the allosteric activation of DNMTs (43). The induction of DNA hypermethylation with drugs, hormones, and other biological compounds has already been observed (42). The other indirect evidence for such a mechanism is the blockage of cancer-specific processes by SAM supplementation, which results in DNA hypermethylation and gene downregulation (44, 45). Taking this into account, it seems reasonable that VPA, either *in vitro* studies or phase I/II clinical trials, induced cell growth inhibition on both benign cells, such as vascular pericytes, cancers, as well as acute myeloid leukemia and solid malignancies (46).

The mechanism of action of VPA, which we propose, is based on allosteric enzyme activation from one side, but also on the oxidative demethylation (hypomethylation) through ROS. That negative association between DNA methylation and oxidative stress was recently confirmed (28). In this paper, we showed that the level of 8-oxo-dG is a good marker of the oxidative stress. As a product of ROS reaction with DNA, we can assume the red-ox state of the cell. The formation of 8-oxo-dG provides information on how deep is the DNA methylation process due to the reaction of the methyl group of m^5^C with ROS.

The most important aspect of our studies is clinical relevance. The meta-analysis of 210 patients with GBM treated with different AEDs (VPA, carbamazepine, phenytoin) showed significantly longer survival than those who were not, and patients treated with VPA had significantly longer survival than those who had received other AEDs (17). However, that work did not specify the kind of chemotherapy that was used in the analyzed patients (whether it was temozolomide or not).

The observation that GBM patients may benefit from VPA therapy, supports our results. We have recently shown that the DNA methylation level in the cell depends on oxidative damage and is reversely correlated with ROS reaction products (28). It is also known that elevated ROS levels in the cell promote tumor development and progression (47). However, the relationship between DNA methylation level with cancer is less obvious. DNA hypermethylation leads to gene down expression including tumor suppressor gene promoters. The methylation of DNA diminishes the affinity of transcriptional factors to the target sequence. On the other hand, global DNA hypomethylation, resulting in total gene expression deregulation, is observed in many tumors (27). For many years, the research focus was directed toward DNA hypomethylating events, and the antineoplastic effect of various drugs was regarded as a consequence of oxidative stress induction and ROS-mediated cell damage (48). However, the efficacy of DNA demethylation agents is limited, and indications are selected (49). The reason for this is the occurrence of global oxidative damage of the cell under such oncological treatment. Surviving cells are highly resistant to any treatment and produce aggressive recurrences. The factors affecting global DNA hypomethylation are recently within the scope of many studies (50, 51), especially because it was proven that the hypermethylated phenotype signifies better survival in glioma (52).

**Figure 7:**
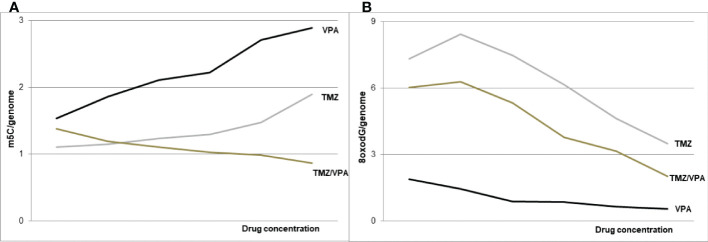
The schematic of VPA and TMZ impact on DNA (m^5^C) methylation – **(A)** and DNA (8-oxo-dG) oxidation – **(B)**, alone and in combination. The concomitant application of the both drugs induces DNA hypomethylation.

The results of our study clearly identify the DNA hypermethylating effect of VPA (**Figure 7**), which can be regarded as the antineoplastic one. The hypomethylation with TMZ in our experiments identifies possible obstacles to the combined therapy of temozolomide, a standard for chemotherapy of primary and recurrent GBM (53), with valproic acid, which is recommended in symptomatic tumor-related epilepsy as a first-line treatment, especially during temozolomide therapy (1). Despite some cell lines studies showing a promising additive effect of VPA on TMZ (54–56), no significant positive impact on overall survival was observed in clinical trials while incorporating the VPA together with TMZ (18). VPA shows the ability to affect TMZ sensitivity in GBM cell lines suggesting that it is the chemosensitizing drug (54). Our experiments show that combined therapy with both drugs leads to total DNA hypomethylation, which suggests the lack of a clearly positive clinical effect of VPA in GBM.

Generally, it turned out that valproic acid acts on two levels of epigenetic cell’s machinery. In addition to histone acetylation, VPA induces reprogramming and increases the total DNA methylation level in glioblastoma cell lines in a dose-dependent manner. The DNA hypermethylation effect of VPA alone can be beneficial for GBM treatment, but not in a combination with TMZ, which induces DNA demethylation (**Figure 7**). Therefore, further clinical trials, are needed to evaluate the combining VPA/TMZ treatment effects.

## Data availability statement

The original contributions presented in the study are included in the article/**Supplementary Material**. Further inquiries can be directed to the corresponding author.

## Author contributions

MZN-B data curation, created the concept of the research, methodology formal analysis, funding acquisition, writing–original draft, writing–review and editing. AMB created the concept of the research, methodology, writing–original draft. AMB planned and carried out experiments, methodology, writing–original draft. IG performed the experiments, project administration. MG-P performed the experiments. All authors contributed to the article and approved the submitted version.

## Funding

This work was supported with funding from the National Science Center Poland (grant nr.: 2020/37/B/NZ5/03249) to MZN-B.

## Conflict of interest

The authors declare that the research was conducted in the absence of any commercial or financial relationships that could be construed as a potential conflict of interest.

## Publisher’s note

All claims expressed in this article are solely those of the authors and do not necessarily represent those of their affiliated organizations, or those of the publisher, the editors and the reviewers. Any product that may be evaluated in this article, or claim that may be made by its manufacturer, is not guaranteed or endorsed by the publisher.
