# Supplementary material for: Cross-reactivity between histone demethylase inhibitor valproic acid and DNA methylation in glioblastoma cell lines
Source: Front Oncol. 2022 Nov 16;12:1033035. doi: 10.3389/fonc.2022.1033035 (PMC9709419; doi:10.3389/fonc.2022.1033035)
Supplement: Supplementary file 1 [file DataSheet_1.pdf]

Supplementary Table 1.

Total 5-methylcytosine (m^5C) contents (R) in DNA of glioblastoma (T98G, U138, U118), cervical cancer (HeLa), and non-neoplastic (HaCaT) cell lines after 3, 12, 24 and 48 hrs (T, time) of incubation in a given VPA concentration [μM]. Numeric data (R) are followed with SD and p values (one-tailed *t*-test, *p < 0,05, **p < 0,01, ***p < 0,001).

VPA	T	T98G			U138			U118			HeLa			HaCaT		
[μM]	[h]	R	SD	p	R	SD	p	R	SD		R	SD	p	R	SD	p
0	3	0,929	0,060		0,744	0,015		0,738	0,036		0,937	0,040		0,717	0,022	
30	3	0,591	0,063	**	0,721	0,055	p>0,05	0,790	0,058	p>0,05	1,092	0,068	**	0,833	0,040	**
50	3	0,691	0,066	**	0,818	0,030	**	0,948	0,031	***	1,297	0,051	***	0,820	0,028	**
100	3	0,816	0,012	*	1,090	0,045	***	1,003	0,056	***	1,350	0,035	***	0,842	0,022	***
250	3	0,975	0,011	p>0,05	1,154	0,081	***	1,136	0,044	***	1,408	0,048	***	0,854	0,030	***
500	3	1,111	0,059	**	1,230	0,028	***	1,207	0,056	***	1,497	0,069	***	0,788	0,072	p>0,05
0	12	0,915	0,065		0,747	0,074		0,725	0,038		0,941	0,040		0,746	0,030	
30	12	0,875	0,036	p>0,05	0,744	0,055	p>0,05	0,785	0,077	p>0,05	1,219	0,043	***	1,057	0,026	***
50	12	0,913	0,052	p>0,05	0,979	0,048	**	0,934	0,019	***	1,393	0,135	***	1,194	0,017	***
100	12	0,945	0,028	p>0,05	1,214	0,087	***	1,079	0,075	***	1,549	0,080	***	1,180	0,039	***
250	12	1,263	0,028	***	1,266	0,032	***	1,232	0,057	***	1,623	0,072	***	1,204	0,023	***
500	12	1,643	0,082	***	1,312	0,084	***	1,247	0,028	***	1,796	0,097	***	1,069	0,032	***
0	24	0,956	0,061		0,727	0,041		0,695	0,011		0,982	0,047		0,727	0,028	
30	24	1,180	0,024	**	0,662	0,026	*	0,636	0,039	*	1,262	0,052	**	1,152	0,038	***
50	24	1,312	0,084	**	0,767	0,042	p>0,05	0,692	0,031	p>0,05	1,640	0,107	***	1,061	0,030	***
100	24	1,451	0,075	***	0,858	0,030	**	0,822	0,039	***	1,860	0,144	***	1,167	0,014	***
250	24	1,678	0,033	***	0,921	0,016	***	1,000	0,076	***	1,997	0,124	***	1,184	0,045	***
500	24	2,157	0,056	***	1,066	0,056	***	1,231	0,077	***	2,062	0,136	***	1,181	0,026	***
0	48	0,909	0,083		0,769	0,036		0,717	0,013		0,985	0,036		0,758	0,028	
30	48	0,897	0,077	p>0,05	0,762	0,035	p>0,05	0,709	0,053	p>0,05	1,320	0,045	***	1,059	0,022	***
50	48	0,916	0,017	p>0,05	0,865	0,089	*	1,005	0,034	***	1,596	0,110	***	1,047	0,016	***
100	48	1,075	0,007	**	1,016	0,010	***	1,108	0,040	***	1,806	0,099	***	1,031	0,033	***
250	48	1,197	0,047	**	1,151	0,085	***	1,210	0,049	***	1,851	0,033	***	0,934	0,067	**
500	48	1,332	0,051	***	1,218	0,087	***	1,286	0,084	***	1,883	0,023	***	0,929	0,044	**
