# Supplementary material for: Cross-reactivity between histone demethylase inhibitor valproic acid and DNA methylation in glioblastoma cell lines
Source: Front Oncol. 2022 Nov 16;12:1033035. doi: 10.3389/fonc.2022.1033035 (PMC9709419; doi:10.3389/fonc.2022.1033035)
Supplement: Supplementary file 2 [file DataSheet_2.pdf]

Supplementary Table 2.

Total 5-methylcytosine (m^5C) contents (R) in DNA of glioblastoma (T98G, U138, U118), and non-neoplastic (HaCaT) cell lines after 3, 12, 24 and 48 hrs (T, time) of combined treatment with TMZ and VPA in given concentrations [μM]. Numeric data given in headings represent R, SD and p values respectively (one-tailed *t*-test, *p < 0,05, **p < 0,01, ***p < 0,001).

		**T98G**				**U138**				**U118**				**HaCaT**			
**T [h]**	**TMZ[μM]** / **VPA [μM]**	**0**	**1**	**30**	**100**	**0**	**1**	**30**	**100**	**0**	**1**	**30**	**100**	**0**	**1**	**30**	**100**
3	**0**	0,72±0,03	0,74±0,03<br>p>0,05	0,76±0,03<br>*	0,79±0,02<br>**	0,72±0,07	0,74±0,00<br>p>0,05	0,79±0,08<br>p>0,05	0,84±0,01<br>*	0,68±0,03	0,68±0,04<br>p>0,05	0,72±0,03<br>*	0,76±0,03<br>**	0,75±0,05	0,82±0,02<br>*	0,89±0,05<br>**	0,99±0,03<br>***
3	**50**	0,72±0,01<br>p>0,05	0,66±0,01<br>**	0,62±0,03<br>**	0,56±0,02<br>***	0,82±0,01<br>*	0,76±0,02<br>p>0,05	0,69±0,01<br>p>0,05	0,64±0,02<br>p>0,05	0,89±0,05<br>***	0,78±0,02<br>***	0,64±0,05<br>p>0,05	0,43±0,05<br>***	0,79±0,02<br>p>0,05	0,82±0,02<br>p>0,05	0,83±0,05<br>p>0,05	0,84±0,07<br>p>0,05
3	**200**	0,79±0,02<br>**	0,64±0,03<br>**	0,56±0,01<br>***	0,39±0,02<br>***	0,98±0,02<br>**	0,86±0,04<br>**	0,64±0,03<br>p>0,05	0,59±0,01<br>*	1,05±0,03<br>***	0,81±0,02<br>***	0,65±0,04<br>p>0,05	0,44±0,01<br>***	0,80±0,03<br>p>0,05	0,82±0,04<br>p>0,05	0,82±0,01<br>p>0,05	0,84±0,08<br>p>0,05
3	**350**	0,88±0,01<br>***	0,53±0,02<br>***	0,42±0,01<br>***	0,28±0,01<br>***	1,02±0,04<br>***	0,81±0,02<br>*	0,60±0,02<br>*	0,54±0,01<br>*	1,18±0,08<br>***	0,95±0,03<br>***	0,53±0,01<br>***	0,39±0,05<br>***	0,82±0,01<br>p>0,05	0,80±0,03<br>p>0,05	0,81±0,04<br>p>0,05	0,82±0,03<br>p>0,05
24	**0**	0,72±0,03	0,71±0,07<br>p>0,05	0,76±0,05<br>p>0,05	0,82±0,03<br>**	0,76±0,03	0,83±0,02<br>**	0,88±0,01<br>***	0,96±0,03<br>***	0,62±0,01	0,67±0,05<br>p>0,05	0,72±0,01<br>***	0,76±0,02<br>***	0,71±0,02	0,82±0,03<br>***	0,91±0,05<br>**	0,98±0,01<br>***
24	**50**	0,74±0,03<br>p>0,05	0,66±0,03<br>**	0,62±0,03<br>**	0,56±0,01<br>***	0,85±0,05<br>*	0,72±0,04<br>p>0,05	0,58±0,02<br>***	0,52±0,07<br>**	0,77±0,01<br>***	0,67±0,04<br>*	0,59±0,03<br>p>0,05	0,55±0,02<br>***	0,76±0,03<br>*	0,78±0,01<br>**	0,86±0,03<br>***	0,94±0,09<br>**
24	**200**	0,86±0,02<br>***	0,66±0,04<br>*	0,60±0,01<br>**	0,56±0,01<br>***	0,89±0,04<br>***	0,81±0,02<br>*	0,79±0,03<br>p>0,05	0,73±0,07<br>p>0,05	0,99±0,03<br>***	0,76±0,01<br>***	0,62±0,02<br>p>0,05	0,42±0,04<br>***	0,78±0,02<br>**	0,87±0,03<br>***	0,98±0,02<br>***	1,00±0,07<br>***
24	**350**	1,16±0,03<br>***	0,72±0,03<br>p>0,05	0,53±0,02<br>***	0,51±0,02<br>***	0,93±0,04<br>***	0,76±0,04<br>p>0,05	0,72±0,02<br>*	0,63±0,09<br>*	1,08±0,08<br>***	0,68±0,02<br>**	0,44±0,03<br>***	0,33±0,02<br>***	0,72±0,03<br>**	0,90±0,02<br>***	0,94±0,02<br>***	0,84±0,02<br>***
48	**0**	0,72±0,03	0,71±0,02<br>p>0,05	0,69±0,04<br>p>0,05	0,69±0,03<br>p>0,05	0,72±0,03	0,74±0,03<br>p>0,05	0,80±0,02<br>**	0,84±0,03<br>***	0,62±0,01	0,54±0,04<br>*	0,51±0,02<br>***	0,44±0,03<br>***	0,71±0,03	0,76±0,02<br>*	0,82±0,03<br>***	0,88±0,09<br>*
48	**50**	0,76±0,03<br>*	0,80±0,02<br>**	0,71±0,05<br>p>0,05	0,69±0,03<br>p>0,05	0,81±0,02<br>**	0,89±0,07<br>**	0,97±0,02<br>***	0,98±0,05<br>***	0,97±0,02<br>***	0,76±0,06<br>**	0,64±0,02<br>p>0,05	0,61±0,03<br>p>0,05	0,78±0,02<br>**	0,78±0,05<br>*	0,82±0,01<br>***	0,86±0,03<br>***
48	**200**	0,83±0,04<br>**	0,72±0,02<br>p>0,05	0,64±0,02<br>**	0,62±0,01<br>**	0,98±0,07<br>**	0,96±0,02<br>***	0,88±0,08<br>*	0,83±0,10<br>p>0,05	1,12±0,05<br>***	0,91±0,06<br>**	0,76±0,03<br>***	0,63±0,03<br>p>0,05	0,76±0,02<br>*	0,79±0,08<br>p>0,05	0,83±0,04<br>***	0,85±0,02<br>***
48	**350**	0,87±0,04<br>***	0,64±0,02<br>**	0,56±0,04<br>***	0,51±0,04<br>***	1,06±0,05<br>***	0,95±0,05<br>***	0,78±0,03<br>*	0,58±0,02<br>***	1,21±0,02<br>***	0,84±0,05<br>**	0,59±0,08<br>p>0,05	0,55±0,02<br>***	0,74±0,04<br>p>0,05	0,81±0,02<br>***	0,86±0,01<br>***	0,82±0,05<br>*
